# Actionable cancer vulnerability due to translational arrest, p53 aggregation and ribosome biogenesis stress evoked by the disulfiram metabolite CuET

**DOI:** 10.1038/s41418-023-01167-4

**Published:** 2023-05-04

**Authors:** Dimitris C. Kanellis, Asimina Zisi, Zdenek Skrott, Bennie Lemmens, Jaime A. Espinoza, Martin Kosar, Andrea Björkman, Xuexin Li, Stefanos Arampatzis, Jirina Bartkova, Miguel Andújar-Sánchez, Oscar Fernandez-Capetillo, Martin Mistrik, Mikael S. Lindström, Jiri Bartek

**Affiliations:** 1 Science for Life Laboratory, Division of Genome Biology, Department of Medical Biochemistry and Biophysics, Karolinska Institutet, S-171 21 Stockholm, Sweden; 2 Institute of Molecular and Translational Medicine, Faculty of Medicine and Dentistry, Palacky University, Olomouc, Czech Republic; 3 Danish Cancer Society Research Center, DK-2100 Copenhagen, Denmark; 4 Pathology Department, Complejo Hospitalario Universitario Insular, Las Palmas, Gran Canaria Spain; 5 Genomic Instability Group, Spanish National Cancer Research Centre (CNIO), Madrid, 28029 Spain

**Keywords:** Cancer, Cell biology

## Abstract

Drug repurposing is a versatile strategy to improve current therapies. Disulfiram has long been used in the treatment of alcohol dependency and multiple clinical trials to evaluate its clinical value in oncology are ongoing. We have recently reported that the disulfiram metabolite diethyldithiocarbamate, when combined with copper (CuET), targets the NPL4 adapter of the p97VCP segregase to suppress the growth of a spectrum of cancer cell lines and xenograft models in vivo. CuET induces proteotoxic stress and genotoxic effects, however important issues concerning the full range of the CuET-evoked tumor cell phenotypes, their temporal order, and mechanistic basis have remained largely unexplored. Here, we have addressed these outstanding questions and show that in diverse human cancer cell models, CuET causes a very early translational arrest through the integrated stress response (ISR), later followed by features of nucleolar stress. Furthermore, we report that CuET entraps p53 in NPL4-rich aggregates leading to elevated p53 protein and its functional inhibition, consistent with the possibility of CuET-triggered cell death being p53-independent. Our transcriptomics profiling revealed activation of pro-survival adaptive pathways of ribosomal biogenesis (RiBi) and autophagy upon prolonged exposure to CuET, indicating potential feedback responses to CuET treatment. The latter concept was validated here by simultaneous pharmacological inhibition of RiBi and/or autophagy that further enhanced CuET’s tumor cytotoxicity, using both cell culture and zebrafish in vivo preclinical models. Overall, these findings expand the mechanistic repertoire of CuET’s anti-cancer activity, inform about the temporal order of responses and identify an unorthodox new mechanism of targeting p53. Our results are discussed in light of cancer-associated endogenous stresses as exploitable tumor vulnerabilities and may inspire future clinical applications of CuET in oncology, including combinatorial treatments and focus on potential advantages of using certain validated drug metabolites, rather than old, approved drugs with their, often complex, metabolic profiles.

## Introduction

Recent technological advances combined with sophisticated computational algorithms have facilitated the discovery of new compounds of clinical value [1]. Nonetheless, drug development remains a costly and time-consuming process, circumstances which make drug repurposing an attractive alternative strategy to battle human diseases. To this effect, we have previously elucidated the mechanism for how disulfiram (DSF), commonly used as an alcohol-aversion drug, kills cancer cells and provided epidemiological evidence supporting the notion of DSF’s repurposing for cancer therapy [2]. We showed that DSF´s anticancer activity relies on its copper-containing metabolite (CuET) that triggers proteotoxic stress through NPL4 sequestration, thereby crippling the p97-dependent protein turnover pathway followed by induction of the unfolded protein response (UPR) [2].

Aberrant protein homeostasis leads to the formation of aggregates with various outcomes for the cancer cell fate and, consequently, cancer therapy. For example, we have previously shown that CuET-induced protein aggregates entrap the ATR kinase resulting in DNA replication stress and the associated synthetic lethality in BRCA1/2 deficient cells [3, 4]. Moreover, endocrine treatment triggers aggregates of various compositions in breast cancer cells, and their analysis may aid the identification of drug-resistant cells [5]. However, aggregate formation is not restricted to cells treated with chemicals. Mutant p53 isoforms can form aggregates with dominant-negative effects over the wild type (wt) p53 and its paralogs [6], thereby potentially impacting tumor progression and responses to treatment.

Both proteotoxic stress and the UPR trigger a feedback mechanism known as the integrated stress response (ISR) that controls the intracellular protein load through translational reprogramming [7]. The key step in ISR is eIF2a phosphorylation followed by global translation suspension with residual ribosomal activity allowing selective translation of mRNAs that either ameliorate the proteotoxic stress or induce cell death [8]. P53 impacts translation by fine-tuning ribosome biogenesis (RiBi), modulating transcription of 4E-BP1 (master regulator of translation) [9], and shaping the formation of translation initiation complexes (ternary, eIF4F) [10]. Inspired by our initial observation that both disulfiram and CuET enhance p53 protein level and activate UPR signaling including phosphorylation of eIF2 [2], here we set off to investigate the mechanistic links of CuET with protein translation, ribosome biogenesis, and p53. We furthermore wished to elucidate the temporal order of events that occur in human cancer cells exposed to CuET, and their consequences with potential relevance for tumor therapy. Our experiments and results obtained while pursuing these topics are described below, including our discovery of an unexpected mode of p53 inactivation in CuET-treated tumor cells. Furthermore, together with rapid and robust CuET-evoked impairment of ribosome function and the ensuing impact on ribosome biogenesis and autophagy, these striking phenotypes led us to propose, and successfully validate, an actionable vulnerability of cancer cells that could be exploited in future treatment strategies.

## Results

### CuET treatment suppresses translation in an ISR-dependent manner

**Fig. 1:**
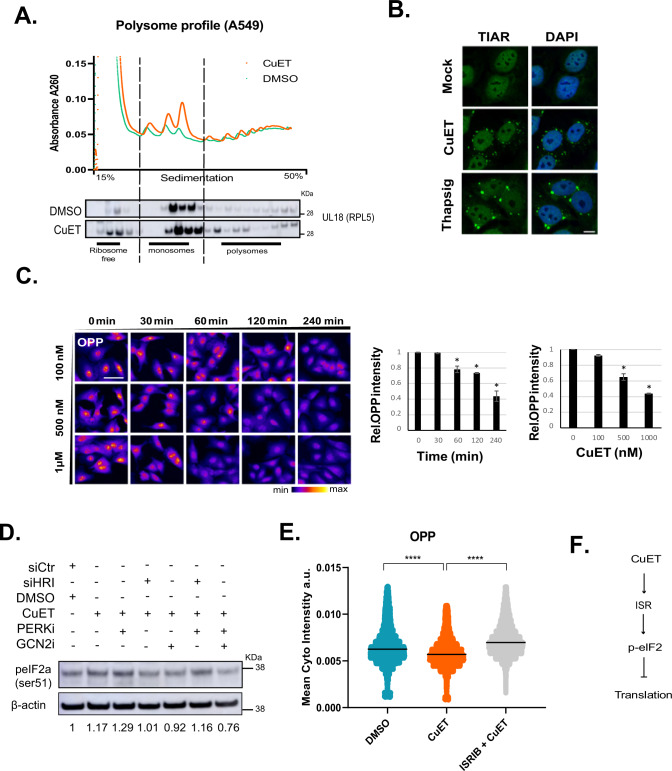
CuET rapidly blocks protein synthesis. **A** Upper panel: Polysome profiling of A549 epithelial cells following treatment with CuET for 1 h, lower panel: immunoblotting of the ribosomal protein RPL5 (uL18) in polysome profile fractions of A549 under the same experimental conditions. **B** Representative IF images of TIAR-containing stress granules after CuET treatment of A549 cells. The UPR inductor thapsigargin was used as a positive control. scale bar = 10 μm. **C** O-propargyl-puromycin (OPP) incorporation followed by high-content microscopy for the quantitation of translation rates in A549 cells treated with CuET or DMSO. 750–1500 cells were analyzed per experiment (data are shown as mean ± SD, *n* = 3 biological replicates, *p < 0.05) scale bar: 50 µm. **D** Immunoblotting of phosphorylated eIF2a (ser51) following treatment of A549 with CuET (2 h) +/–PERK inhibitor combined with siRNA against HRI or chemical inhibition of GCN2. Numbers below the blot indicate the signal ratio p-eIF2a/β-actin. **E** OPP incorporation followed by high-content microscopy for the quantitation of translation rates in A549 cells treated with CuET (2 h) +/− the ISR inhibitor ISRIB. 1000–2000 cells were analyzed per experiment (data shown as mean ± SD, *n* = 3 biological replicates, ****p < 0.001). **F** Schematic model connecting CuET treatment and translation.

To address any potential impact of CuET on ribosome function, we first treated human lung carcinoma A549 cells with CuET (1 μM, 1 h) and utilized polysome profiling to examine the effects on global translation. As shown in Fig. 1A, CuET treatment resulted in higher monosome peaks (upper panel), while elevating RPL5 (ul18) levels in monosome- and ribosome-free fractions (lower panel), both indicative of translation attenuation. Using TIAR (encoded by the *TIAL1* gene) as a marker of translational arrest-induced stress granules, it became evident that CuET behaves similarly to thapsigargin (Fig.1B and Supplementary Fig. 1A), a known attenuator of translation due to elevated ER stress and UPR signaling [11]. Higher monosome peaks may reflect a shift of the translational output from polysome-fueled to monosome-fueled without affecting overall translation rates [12]. To test this possibility, we used click-it chemistry with O-propargyl-puromycin (OPP, Fig. 1C and Supplementary Fig. 1B) or L-azidohomoalanine (AHA, Supplementary Fig. 1C) incorporation followed by high-content microscopy. We found that CuET rapidly blocks protein production in a dose- and time-dependent manner. ISR is mediated by various kinases that converge on eIF2a phosphorylation in order to block translation [13]. Excluding PKR which is involved in biological responses to viruses, we then explored the effect of three ISR-related kinases (PERK, HRI, and GCN2) on eIF2a following the CuET treatment. We found that simultaneous inhibition of PERK and GCN2 (Fig. 1D) or administration of an ISR inhibitor (Fig. 1E) rescued the negative effect of CuET on translation, as shown by either phosphorylation of eIF2a or OPP incorporation followed by high-content microscopy. Taken together, these findings support the notion that ISR-mediated translational arrest is a previously unrecognized and robust phenotype that occurs very early in human cancer cells exposed to CuET treatment (Fig. 1F).

### Treatment with CuET reshapes the nucleolar structure

**Fig. 2:**
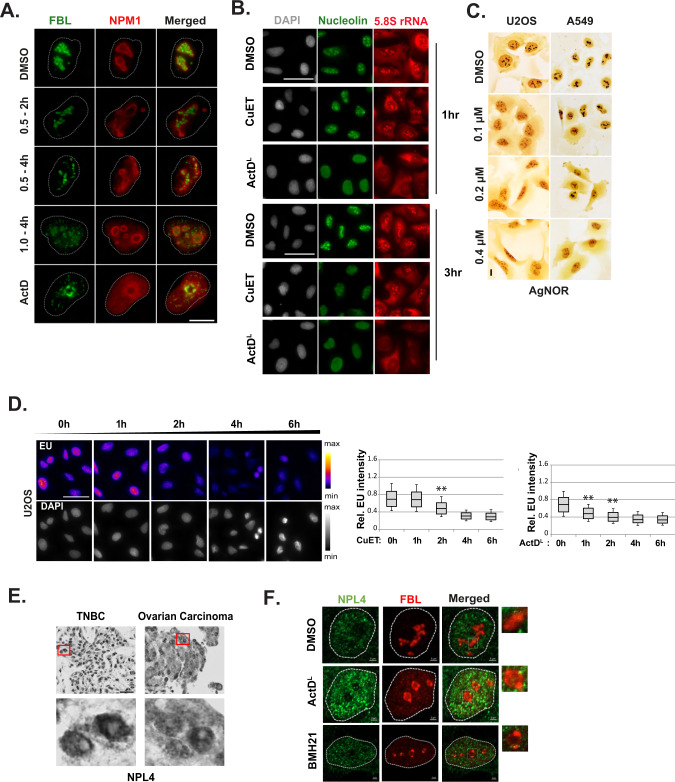
CuET alters the nucleolar morphology. **A** Representative IF images of A549 nuclei treated with CuET or ActD^L^ for the indicated time points. Fibrillarin (FBL) or nucleophosmin (NPM1) were used as nucleolar markers. Scale bar: 5 μM. **B** Representative IF images of nucleolin and 5.8S rRNA levels in A549 cells treated with CuET or ActD^L^ for the indicated time points. Scale bar: 50 µm. **C** AgNOR staining of U2OS or A549 cells following a four-hour treatment of increasing CuET doses. Scale bar: 10 μm. **D** Ethylene uridine (EU) levels were calculated following IF and high content imaging of U2OS treated with 1 μM CuET for the indicated time points. 750–1500 cells were analyzed per experiment (data are shown as mean ± SD, *n* = 3 biological replicates, **p < 0.01). Scale bar: 50 µm. **E** Detection of NPL4 protein levels with immunocytochemistry in samples from patients with triple-negative breast cancer (TNBC) or ovarian carcinoma. Regions in red squares are presented magnified in the bottom panel. Scale bar: 100 μM. **F** Representative IF images of nucleolar structure in U2OS treated with ActD^L^ or BMH-21 as nucleolar stress inducers. Fibrillarin (FBL) was used as a nucleolar marker. Insets depict magnifications of the regions designated in squares. Scale bar: 2 μM.

Perturbed translation may alter RiBi [14] and drive nucleolar stress often manifested by nucleolar reorganization [15]. Using immunofluorescence (IF) to examine fibrillarin (FBL), nucleophosmin (NPM1), and nucleolin as nucleolar markers, we found that CuET alters nucleolar morphology, particularly after prolonged treatments (Fig. 2A, B and Supplementary Fig. 2A). Nucleolar alteration was also verified with silver staining (Fig. 2C) of human A549 or U2OS cells treated with CuET in a dose-dependent manner. These results indicated defective rDNA transcription, and in order to explore this possibility, we utilized a click-it EU incorporation assay combined with high-content microscopy. We noticed that prolonged CuET treatment caused a drop in transcription levels comparable to those resulting from exposure to a low dose ActD (ActD^L^), a drug known to block RNA pol I transcription [16] (Fig. 2D). Nevertheless, quantitation of 5.8S rRNA with IF (Supplementary Fig. 2A) or 47S rRNA with qRT-PCR (Supplementary Fig. 2B) did not show any significant changes between cells treated with CuET or control DMSO as a vehicle, respectively. Moreover, the CuET-induced structural changes in nucleoli differ from those observed under ActD^L^ treatment which is known to induce the formation of nucleolar caps that indicate arrest of RNA pol I-mediated transcription (Fig. 2A and Supplementary Fig. 2A) [16]. Interestingly, we found that CuET’s molecular target, NPL4, resides outside of nucleoli of both, human cells in tissue sections from clinical tumor specimens, and cultured cancer cell lines treated with pol I chemical inhibitors (Fig. 2E, F and Supplementary Fig. 2C). The immunohistochemical analyses of NPL4 subcellular localization were performed on archival paraffin sections from two complementary cohorts of: i) triple-negative breast cancer (*n* = 63), and ii) serous ovarian carcinomas (*n* = 51), with all these 114 human clinical samples showing consistently NPL4 protein localized outside the nucleolus. Together, these complementary results obtained with cultured cell lines and clinical specimens excluded nucleoli as the primary cellular regions of action for CuET. Moreover, the lack of the impaired ribosome biogenesis complex (IRBC) [17] to bind MDM2 and thereby stabilize and increase the abundance of p53 (Supplementary Fig. 2D, E) following CuET treatment suggested an atypical nucleolar stress scenario, distinct from the one commonly seen in response to chemical inhibition of RNA pol I. These findings suggested a delayed, possibly indirect inhibitory effect of CuET on RNA pol I-mediated transcription, and raised a question about the fate of p53 in CuET-treated cells.

### CuET triggers p53 entrapment in NPL4-containing aggregates

**Fig. 3:**
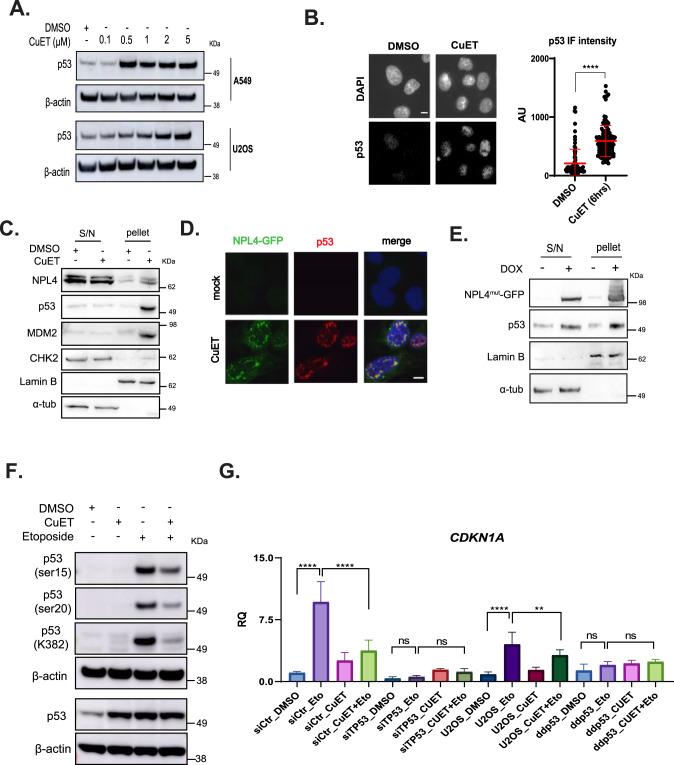
CuET triggers p53 entrapment in NPL4-rich aggregates. **A** Immunoblotting of p53 protein levels following increasing concentrations of CuET in U2OS or A549 cells. **B** IF-based quantitation of p53 levels following CuET treatment of A549 cells. Data are shown as mean ± SD, *n* = 3 biological replicates, ****p < 0.001. Scale bar: 10 μM. **C** NPL4, p53, MDM2, and CHK2 protein levels following CuET treatment and fractionation of A549 cells. Lamin B and α-tubulin were used as markers for the soluble (S/N) and insoluble (pellet) fractions, respectively. **D** Representative IF images of GFP-tagged NPL4 and p53 protein levels following treatment of NPL4-GFP U2OS cells with CuET. Scale bar: 10 μM**. E** NPL4 and p53 protein levels in U2OS cells ectopically expressing NPL4^mut^. Lamin B and α-tubulin were used as markers for the soluble (S/N) and insoluble (pellet) fractions, respectively. **F** Immunoblotting of various p53 post-translational modifications (PTM) in A549 cells treated with CuET+/− (6 h) Etoposide (last 2 h). Etoposide was used as a positive control known to induce activating p53 PTMs. **G** *CDKN1A* mRNA levels following treatment with CuET (6 h) +/− Etoposide (last 2 h). Two different cell models were used: A549 transfected with siRNA against *TP53* or control siRNA and U2OS cells compared to U2OS cells carrying a dominant negative p53 mutant (ddp53) (data are shown as mean ± SD, *n* = 3 biological replicates, ****p < 0.001, ***p = 0.01, **p < 0.01, ns non-significant).

We previously reported that CuET induces a higher p53 protein level in human cells irrespective of their p53 genetic status [2], an observation that prompted us to investigate p53 homeostasis upon CuET treatment of human cells further. We initially found that CuET exposure causes p53 accumulation in a time- and dose-dependent manner, in both U2OS and A549 cells, as shown by immunoblotting and immunofluorescence (Fig. 3A, B, and Supplementary Fig. 3A). Given the above-mentioned inability of CuET-treated cells to trigger the p53-mediated checkpoint that normally responds to aberrant RiBi, we next assessed whether p53 might be subject to some mechanism that renders p53 dysfunctional. As CuET entraps several proteins into NPL4-containing aggregates [2, 3], we explored the potential presence of p53 in such formations. Following CuET treatment and fractionation of either U2OS or A549 cells, we indeed detected p53 in the insoluble cellular fractions (Fig. 3C and Supplementary Fig. 3B). Interestingly, MDM2, but not CHK2 (both known p53 interactors [18, 19]) was also found in the insoluble fractions (Fig. 3C and Supplementary Fig. 3B) indicating specific entrapment of the p53-MDM2 complex in the aggregates. Our findings were further corroborated by the observation that p53 accumulates in NPL4-rich subcellular regions, as shown by IF analysis of U2OS cells engineered to express GFP-tagged NPL4 (Fig. 3D). Moreover, ectopic expression of a mutant NPL4 that spontaneously forms aggregates even without CuET exposure [2] also caused accumulation of p53 in NPL4-rich insoluble cellular fractions (Fig. 3E and Supplementary Fig. 3C). To test the possibility that CuET induced p53 elevation due to the previously reported CuET-induced replication stress and DNA damage [3], we treated A549 cells with small chemical inhibitors against the three central DNA damage response kinases: ATM, ATR, and DNA-PK. Despite such DNA damage signaling inhibition, however, we could not reverse the effect of CuET on p53 accumulation (Supplementary Fig. 3D–F).

These results were consistent with the scenario in which CuET entraps p53 and MDM2 in NPL4 protein aggregates, thereby undermining p53 turnover and likely leading to functional inactivation of such sequestered p53 protein over time. Our working hypothesis that the CuET-triggered aggregation may render p53 largely inactive raised two important predictions, namely that: i) the otherwise functionally important activatory posttranslational modifications of p53 could be absent or robustly reduced, and ii) transcriptional activity of p53 towards promoters of its target genes would be much lower after treatment with CuET. These two predictions were then experimentally tested and both were indeed confirmed. First, immunoblotting analysis of a series of well-established post-translational modifications of p53 [20], including phosphorylation of serine residues 15 and 20, as well as acetylation of lysine 382, were not only uninduced after treatment by CuET alone but these modifications that are otherwise highly induced after etoposide treatment were markedly reduced when etoposide was added to cells treated by CuET for 6 h (Fig. 3F) or 3h (Supplementary Fig. 3G), respectively. Second, monitoring the expression kinetics of a prominent p53 target gene, *CDKN1A* by quantitative real-time PCR in the two p53 wild-type cell lines, A549 and U2OS, again treated alone by CuET or etoposide, respectively, or both compounds combined, showed that the transcriptional activity of p53 in CuET-treated cells was considerably compromised, leading to robust reduction of *CDKN1A* transcripts induced by etoposide in both models (Fig. 3G). These results were also consistent with the expression pattern of MDM2, another canonical p53 target gene, whose etoposide-induced expression in the A549 model was effectively eliminated when such cells were also treated by CuET (Supplementary Fig. 3H). Furthermore, the modest expression of CDKN1A seen after treatment with CuET alone was either entirely (U2OS cells) or partly (A549 cells) independent of p53, as documented by the parallel analysis of cells with experimental silencing of endogenous p53 by either RNA interference (in A549 cells) or inducible expression of a dominant-negative C-terminal p53 fragment capable of binding to and inhibiting the endogenous WT p53 (ddp53, in U2OS cells) (Fig. 3G).

Taken together, these results document that treatment by CuET leads to the entrapment of endogenous p53 protein in the NPL4 aggregates, resulting in robustly impaired post-translational modifications and compromised transcriptional activity of p53. Functional consequences and mechanistic interpretation of these results are presented in the Discussion section.

### CuET synergizes with pol I and autophagy inhibitors in inducing p53-independent cell death

**Fig. 4:**
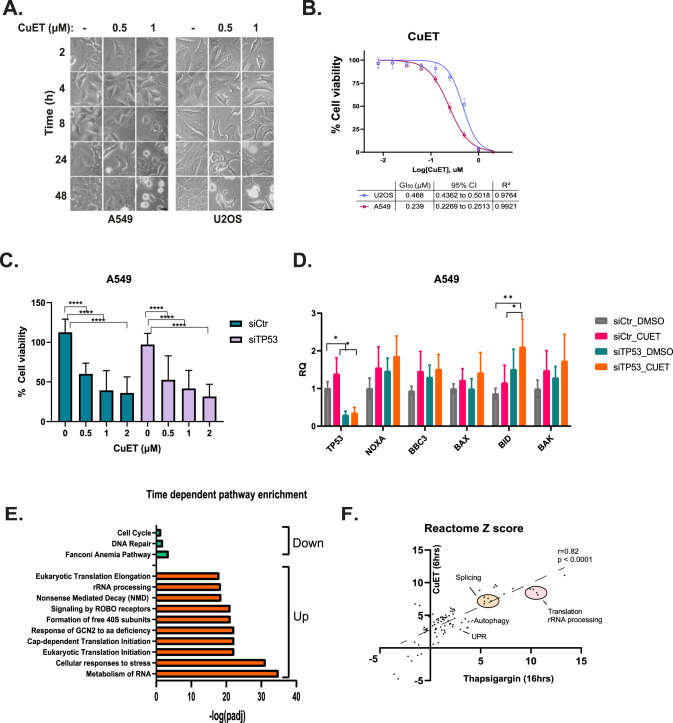
CuET induces cell death in a p53-independent manner. **A**. Representative light microscope images following treatment of A549 or U2OS cells with various concentrations of CuET for different time points. Scale bar: 10 μM**. B** CuET dose-response curves in A549 and U2OS cells. GI_50_ values are shown in the table with 95% confidence intervals, along with R^2^ values quantifying the goodness of fit (data are shown as mean ± SD, *n* = 3 biological replicates). **C** Cell survival analysis (resazurin) in A549 cells treated with siRNA against *TP53 *+/− various concentrations of CuET for 24 h. **D** Quantitative RT-PCR of various p53 targets in A549 cells treated with CuET (6 h) +/− siRNA against *TP53* (data are shown as mean ± SD, *n* = 3 biological replicates, **p < 0.01, *p < 0.05, non-significant values are not shown). **E** Barplot of pathway enrichment terms (Reactome) for genes that are monotonically up-or down-regulated following CuET treatment. **F** Scatterplot depicting the correlation of Reactome terms among DE genes affected by CuET or thapsigargin.

CuET triggers cell death in a dose- and time-dependent manner (Fig. 4A), with different cell lines showing variable and distinct sensitivities to such treatment (Fig. 4B). To further explore the degree of (in)dependency of the CuET-induced cell death on p53 function, we treated p53-proficient and *TP53*-depleted A549 cells with CuET and found that p53 was dispensable for CuET’s capacity to kill these cancer cells (Fig. 4C). This notion also applies to mutant p53 as we have previously shown that cell lines carrying either wt or mutant p53 show similar sensitivity to CuET [2] (Supplementary Fig. 4A). Moreover, CuET was found to induce the expression of various apoptotic genes in cells depleted of p53 further supporting p53-independent mechanisms behind the observed CuET-triggered cell death (Fig. 4D)

To provide insights into potential additional gene dependencies that mediate CuET’s cytotoxicity, we performed RNA-Seq analysis in A549 cells treated with CuET for 2 or 6 h (Supplementary Fig. 4B and Supplementary Table S1a,b) and compared the expression profile of DE genes to the expression of genes correlating with the efficacy of disulfiram (DepMap, 21Q2, PRISM 19Q4, Supplementary Table S1c). Strikingly, we found that metallothionein-encoding genes (e.g., *MT1E*, *MT2A*), known to protect cells from disulfiram cytotoxicity [21], were significantly upregulated following CuET treatment of A549 cells (Supplementary Fig. 4C), indicating a likely negative feedback loop that may eventually lead to treatment resistance. This is consistent with our recent findings regarding the counteracting effect of cannabidiol in the anticancer activity of CuET due to the induction of metallothioneins [22].

Next, to further explore cellular mechanisms following the CuET-mediated translational arrest and nucleolar stress, we examined the time-dependent alterations in gene expression using our RNA-Seq data. Genes were clustered in six groups according to the mode in their expression changes (up- or down-regulated, Supplementary Fig. 4D Supplementary Table S1d). DE genes were then subjected to pathway enrichment analysis using either the monotonically upregulated (clusters 1, 3, 6) or the monotonically downregulated genes (clusters 4, 5). Figure 4E shows that genes that are monotonically suppressed are related to DNA damage response and cell cycle, while genes with monotonic upregulation are found in groups associated with protein metabolism (Supplementary Table S1e,f). Given that CuET treatment did not cause any significant changes in the cell cycle phase distribution in diverse cell lines (Supplementary Fig. 4E, F), we next focused on protein translation and compared our RNA-Seq data to those reported for an ISR mouse model [23] (Supplementary Table S1g–i). While this murine study achieved translation attenuation through the administration of thapsigargin, analysis of their reported transcriptomic data pointed towards upregulation of genes implicated in translation [23], similar to our present results (Fig. 4F). Such consistent feedback-mediated induction of genes regulating protein metabolism led us to speculate that simultaneous inhibition of pol I and CuET treatment could mutually strengthen their respective cytotoxic effects.

**Fig. 5:**
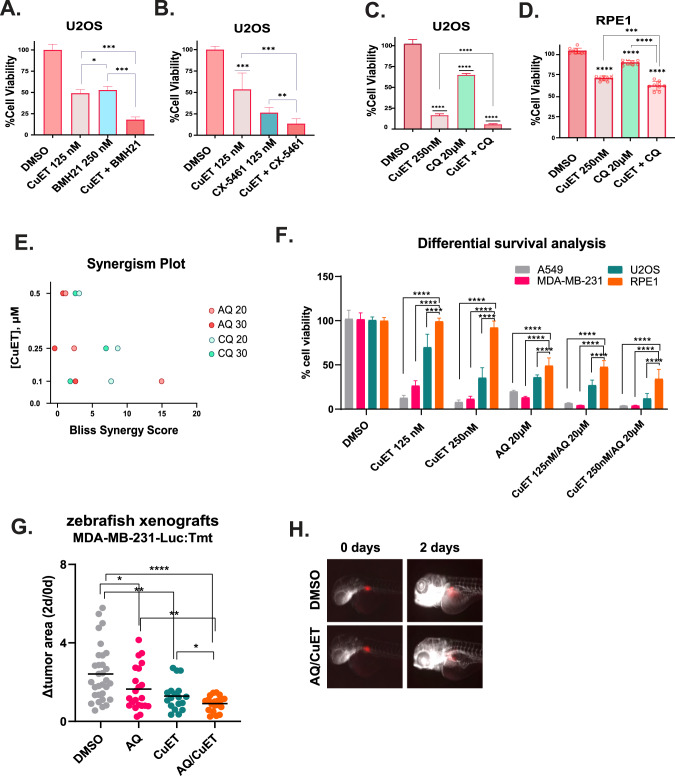
RiBi and autophagy inhibition potentiate the cytotoxic effect of CuET. **A** Survival analysis of U2OS cells treated with the synergistic pairs of CuET, BMH-21, or their combination (data are shown as mean ± SD, *n* = 3 biological replicates, ***p = 0.01, *p < 0.05). **B** Survival analysis of U2OS cells treated with the synergistic pairs of CuET, CX-5461, or their combination (data are shown as mean ± SD, *n* = 3 biological replicates, ***p = 0.01,**p < 0.01). **C**, **D** Survival analysis of U2OS (**C**) or RPE1 (**D**) cells treated with the synergistic pairs of CuET, chloroquine (CQ), or their combination (data are shown as mean ± SD, *n* = 3 biological replicates, ****p < 0.001, ***p = 0.01). **E** Comparative plot showing the Bliss synergy scores of the dose pairs tested among combinations of CuET/AQ (amodiaquine) and CuET/CQ. **F** Differential survival analysis between three cancer cell lines (A549, U2OS, MDA-MB-231) and RPE1 treated with CuET+/− AQ (data are shown as mean ± SD, *n* = 3 biological replicates, ****p < 0.001). **G** Quantification of the transplanted fluorescent tumor area in zebrafish xenografts treated with DMSO vehicle, AQ, CuET, or their combination for 48 h. The end-point signal (2 days) is normalized to the initial one (0 days) for each sample tested (Δtumor area). **H** Representative IF images of xenografts treated with DMSO or the combination of AQ/CuET. Tumors composed of transplanted MDA-MB-231 cells are shown in red.

To test this hypothesis, we next analyzed post-treatment cell survival, combining CuET with either BMH-21 or CX-5461, two compounds known for their pol I inhibitory activity [24]. While such analysis revealed a greatly enhanced cytotoxic impact when using certain drug doses in both compound dose matrices, the BMH-21/CuET combination displayed a more robust cytotoxicity pattern than CuET combined with CX-5461, implying that the DNA-damaging activity of CX-5461 could potentially antagonize CuET’s cytotoxicity in certain dose ratios in the nanomolar range (Fig. 5A, B, and Supplementary Fig. 5A–D).

Another aspect of our transcriptomic analysis was that CuET induced the expression of autophagy-related genes. Autophagy is induced in response to ISR activation [13] and upon ribosomal stress [25] and promotes resistance to various cancer therapies [26]. We, therefore, asked whether autophagy inhibition could enhance the cytotoxicity of CuET, by preventing cancer cells from evading CuET’s anticancer effect. To this end, we combined CuET with Chloroquine (CQ), an antimalarial drug known to inhibit autophagy flux by impairing autophagosome-lysosome fusion [27]. Cell survival assays showed that CQ indeed potentiated the cytotoxic effect of CuET mainly on cancer cells (Fig. 5C, D and Supplementary Fig. 5E, F), indicating that cancer cells may exploit autophagy as a pro-survival mechanism under treatment with CuET, in agreement with previously published data [25].

Based on these results, we argued that combining CuET with concomitant inhibition of pol I and/or autophagy could be a highly effective strategy to undermine cancer cell survival. To validate this rationale while maintaining a reasonable therapeutic window, we combined CuET with the FDA-approved drug amodiaquine (AQ), a compound with dual anticancer activity [28]. AQ is another antimalarial drug structurally related to CQ, which we previously reported to concomitantly inhibit autophagy and RNA pol I, the latter through a mechanism reminiscent of the RNA pol I inhibitor BMH-21 [28]. Our present results showed that the AQ/CuET combination offers the highest synergistic scores and could trigger the most potent cancer cell death-inducing effect (Fig. 5E, F and supplementary Fig. 5G, H). Notably, noncancerous human diploid epithelial cells (RPE1) showed greater tolerance to the same combinatorial treatments (Fig. 5F and supplementary Fig. 5I), which might reflect lower basal translational activity and more modest levels of CuET-induced NPL4-rich aggregates (supplementary Fig. 5J, K). Such differences may suggest the existence of a potentially actionable therapeutic window that might be exploited clinically. To further support our findings in an in-vivo model system, we utilized a zebrafish xenograft model with human MDA-MB-231 breast cancer cell transplants. We confirmed that each compound alone reduced the tumor size, by 51% (AQ) or 68% (CuET), respectively. Notably, the combination of the two compounds enhanced the cytotoxic activity, as reflected by the reduction of the tumor size by 76% (Fig. 5G, H). Importantly, using internationally established criteria, we found there was zero toxicity towards the fish under the experimental conditions of the combined treatment (Supplementary Table 1j), a result that further supported the cancer-selective effect of the compounds and the feasibility of their tolerated application in vivo. In conclusion, these results indicated that human cancer cells may exploit enhanced RiBi and autophagy to mitigate the cytotoxic effects of CuET treatment, revealing actionable vulnerabilities of cancer cells that may be exploited in oncology.

## Discussion

Reflecting their altered biology, cancer cells commonly experience higher levels of endogenous stresses such as replication stress and genomic instability, proteotoxic and metabolic perturbations, at the same time making cancer more dependent on stress-tolerance pathways for their survival and growth. Such selective dependencies can be exploited in cancer therapy, generally through inhibitors of the stress-adaptation mechanisms or by drugs that further enhance the endogenous stress up to suprathreshold levels that are not tolerated by cancer cells, while largely sparing normal cells. Among repurposed drugs that target and/or enhance such tumor cell vulnerabilities is also the old alcohol-abuse drug disulfiram, more specifically one of its major in vivo metabolites, CuET [2]. The molecular target of CuET is the ubiquitin-binding protein NPL4, a key component of the protein degradation machinery upstream of the proteasome [2]. We have previously reported that CuET-induced aggregation of NPL4 triggers proteotoxic [2] and replication stress [3], the latter also increasing endogenous DNA damage in tumor cells and thereby contributing to CuET’s cytotoxicity. These findings, along with the multiple ongoing clinical trials and hopes for repurposing disulfiram in oncology, have raised further mechanistic questions about CuET, namely what is: i) the full spectrum of CuET’s cellular effects; ii) the temporal order of the cellular phenotypes evoked; and iii) the mechanistic basis of potential resistance to disulfiram/CuET treatment and ways to overcome it? In this study, we have addressed and found some answers relevant to these three outstanding questions, the solution of which may contribute to a better understanding of tumorigenesis, provide new biomarkers to guide clinical trials, and help design strategies to avoid or overcome potential cancer therapy resistance mechanisms.

**Fig. 6:**
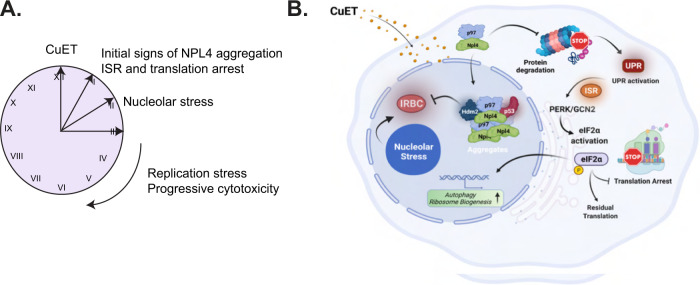
Schematic illustration of the proposed model. **A** Schematic presentation of the temporal order for some key cancer cell phenotypes evoked following CuET treatment. **B** Graphical presentation of the proposed model: the disulfiram metabolite CuET binds NPL4-p97, blocking its role in protein degradation, with ensuing proteotoxic stress and eventually inducing cell death. The NPL4/CuET-nucleated protein aggregates sequester p53 (alongside MDM2) that can no longer be targeted for degradation and thus accumulates, while the p53 activity becomes lower over time, reflecting the progressive aggregate formation and hence p53 sequestration. At the same time, CuET impact activates the cellular ISR and drives the PERK/GCN2a-mediated eIF2a phosphorylation and translational arrest. Cells respond via upregulation of genes affecting, among other mechanisms, RiBi and autophagy, two processes that, when blocked pharmacologically in combination with CuET, show more detrimental effects on cancer cell survival (Figure created with BioRender.com).

First, we report here that CuET exposure evokes a potent arrest of the ribosomal process of translation, a phenotype that follows phosphorylation of eIF2a via ISR kinases known to be activated by aberrant protein metabolism (Fig. 6). This finding is further supported by the fact that the transcriptomic landscape following CuET treatment that we report here resembles thapsigargin-induced ISR in vivo. The prompt protein synthesis blockade that we observed is consistent with translational defects that follow perturbations in ubiquitination [11] such as those one might expect to occur following CuET-mediated neutralization of NPL4 [2]. Another previously unnoticed feature of CuET-treated tumor cells that we identified here is nucleolar restructuring observed later after the translational pausing, with features distinct from the nucleolar stress response to chemical inhibition of pol I [17] (Fig. 6A).

Second, in terms of the temporal sequence of cellular responses, the translational arrest that we found occurs very promptly, as early as one hour post CuET administration, i.e. coinciding with the earliest detectable signs of NPL4 immobilization [2], and before e.g. any detectable increase of DNA damage or massive protein aggregates, events that become first detectable several hours after CuET administration (Fig. 6A). These results identify the protein synthesis arrest as a very sensitive and acute CuET-triggered cellular phenotype, preceding the nucleolar stress (this study) or genotoxic effects [3].

Third, our transcriptome analysis showed that during the sequential responses to the CuET-induced stress(es), tumor cells activate genes related to pro-survival mechanisms such as RiBi and autophagy. These ‘adaptive’ processes could potentially promote cancer cell survival and thereby decrease the clinical efficacy of CuET (Fig. 6B). This notion was therefore examined and validated by concomitant administration of CuET and chemical inhibitors of pol I and/or autophagy in an effort to enhance the cytotoxic activity of CuET. Indeed, our data support a mutually cytotoxicity-enhancing effect of CuET with either pol I inhibitors (e.g. BMH21) or chemicals that block autophagy, such as chloroquine. Even more potent was the combinatorial effect of CuET with amodiaquine, known to inhibit simultaneously RiBi and autophagy [28]. Relevant to any future applications, we have preclinically validated these treatments also in an in-vivo model of human transplants in zebrafish, using the MDA-MB-231 cancer cells derived from a triple-negative breast carcinoma, a tumor type for which standard-of-care treatment modalities are presently unsatisfactory, highlighting an urgent need for identification of alternative, more efficient therapies.

Furthermore, another significant contribution of our present study is the finding of the unexpected trapping and the ensuing transcriptional inactivation of p53. We have previously shown that CuET binds and aggregates NPL4 and thus inactivates the p97 pathways [2], at the same time aggregating also the ATR kinase, thereby leading to a malfunction of the ATR-CHK1 signaling axis and consequently impaired DNA damage response following CuET treatment [3]. Using both immunoblotting of subcellular fractions and IF microscopy, here we extend this notion to p53 and show that together with its master regulator, MDM2, these two proteins become both entrapped in the NPL4-rich domains (Fig. 3). Over time, this progressive process impedes p53 transport to the proteasome, leading to p53 accumulation. Notably, our data also strongly indicate that such entrapment negatively affects p53 function, as shown by the inactive IRBC checkpoint despite the CuET-evoked RiBi stress phenotype, and p53’s inability to induce its canonical transcriptional target, the CDK inhibitor CDKN1A/p21. Mechanistically, we also show that CuET treatment and the ensuing p53 aggregation largely prevent the signaling phosphorylation and acetylation cascades that otherwise provide the multiple activatory post-translational modifications of p53. Given that proteins entrapped in the CuET-triggered aggregates become immobile [2, 3] and surrounded by NPL4 and other insoluble proteins, the robust inhibition of the p53 modifications that we report here most likely reflects the inability of the p53-modifying enzymes (which themselves are largely not trapped in the aggregates, as we show here for the Chk2 kinase known to phosphorylate Ser20 of p53) to access their p53 substrate. We suggest that the combined lack of the p53 post-translational modifications and the immobilization of p53 that prevents the access of p53 to its target promoters, collectively result in the observed inhibition of p53’s transcriptional activity in CuET-treated cells. While a mechanistically distinct aggregation of p53 had previously been shown to affect p53’s anticancer activity [29], CuET demonstrably retains the ability to kill cancer cells, apparently via p53-independent mechanisms. Conceptually, we further speculate that in cancer cells carrying gain-of-function p53 mutants, CuET could have dual anti-cancer activity, captivating mutant p53 in NPL4-rich aggregates while in parallel imposing its p53-independent cytotoxic program.

Last but not least, our present data open new, potentially exploitable avenues for the design of combinatorial treatments in clinical trials complementing those currently ongoing with disulfiram, or directly with CuET should it be later approved for clinical use. In this context, we would like to propose a new idea related to the general concept of drug repurposing, namely the application of active metabolites of FDA-/EMA-approved drugs, rather than the drugs themselves. Using the metabolite would share the safety and desired efficacy previously validated for the generic drug from which the metabolite is derived in vivo while avoiding the potential unwanted effects of other metabolites of the same generic drug. One example of the latter scenario would be the metabolite of disulfiram (distinct from CuET) [30] that inhibits the aldehyde dehydrogenase enzyme and thereby provides the alcohol-aversion effect, as the latter currently prevents combinations of disulfiram with standard-of-care anti-cancer chemotherapeutics that are dissolved in alcohol, and even raises issues with regard to alcohol-based skin disinfectants. Furthermore, using the active metabolite rather than the drug itself would allow for new metabolite-based patents and drug formulations, thereby likely raising interest from pharmaceutical companies, as this would bypass the limitation that is otherwise seen as an obstacle in any broader repurposing of old approved drugs in medicine.

## Materials and methods

### Cell treatment

U2OS osteosarcoma, A549 lung epithelial carcinoma, MDA-MB-231 breast carcinoma, and RPE1 human normal retina epithelial cells were purchased from the American Type Culture Collection (ATCC). Ddp53-U2OS were provided by Jiri Bartek (Danish Cancer Society) The production of GFP-NPL4 U2OS cells was previously described [2]. All cell lines were maintained in DMEM GlutaMax (Gibco) supplemented with 10% FBS (Sigma-Aldrich) and were tested for mycoplasma monthly (Lonza). Cells were treated with 1 μM CuET [2] or 5 nM Actinomycin D (ActD^L^) (Sigma-Aldrich, A1410) unless otherwise stated for the designated periods. For the synergy tests, CX-5461 (Selleckchem, S2684), BMH21 (Sigma-Aldrich, SML1183), Amodiaquine dihydrochloride dehydrate (AQ, Selleckchem, S4589), and Chloroquine diphosphate salt (CQ, Merck, C6628) were used in increasing concentrations as indicated in the figures/legends. ATM (KU-55933, Selleckchem, S1092), ATR (AZ20, Selleckhem, S7050), and DNAPK (KU-57788, Selleckchem, S2638) inhibitors were used at a final concentration of 1 μM for 30 min prior to cell lysis. Neocarzinostatin was used at 0.5 μg/ml and Hydroxyurea (HU) at 1 mM for 12 h. Thapsigargin (Sigma-Aldrich, T9033) was used at 2 μM for 1.5 h, CHX (Sigma-Aldrich, C4859) at 100 μg/ml for the designated time points, and p97 inhibitor, NMS-873 (p97i, Selleckchem, S7285) at 10 μM for 3 h, PERK inhibitor (PERKi, Merck, 516535) and GCN2 inhibitor (GCN2i, MedChemExpress, HY-100877) were used at 10 μM for 30 min. ISRIB (Sigma-Aldrich, SML0843) was used at a final concentration of 50 nM for 1 hr.

### Antibodies

The antibodies used are as follows: rabbit polyclonal Fibrillarin (Abcam, ab5821), mouse monoclonal Nucleophosmin (Abcam, ab10530), rabbit polyclonal Nucleolin (Abcam, ab22758), rabbit polyclonal RPL5 (uL18) (Abcam, ab86863), mouse monoclonal p53 (Abcam, ab1101), rabbit polyclonal phospho-p53 (ser15) (Abcam, ab1431), rabbit polyclonal phospho-p53 (ser20) (Cell signaling, 9287), rabbit monoclonal acetyl-p53 (K382) (Abcam, ab75754), mouse monoclonal p53 (Santa Cruz, sc-126), rabbit monoclonal p21 (Cell Signaling, 2947), mouse monoclonal MDM2 (mixture of 2A9, 4B2 and 4B11 clones, kindly provided by M.Oren), mouse monoclonal MDM2 (SMP14, Santa Cruz, sc-965), mouse monoclonal MDM2 (Millipore, 05-1530), mouse monoclonal CHK2 (H300, Santa Cruz, sc-9064), mouse monoclonal TIAR (Becton Dickinson, 610352), mouse monoclonal beta-actin (Abcam ab6276), rabbit polyclonal phospho-eIF2a (ser51) (Cell Signaling, 9722), rabbit polyclonal phospho-Chk1 (Ser317) (Cell Signaling 2344), mouse monoclonal 5.8S rRNA (Novus Biologicals, NB100-622), rabbit polyclonal NPL4 (Novus Biologicals, NBP1-82166), mouse monoclonal lamin B1 (Santa Cruz, sc-6217), mouse monoclonal a-tubulin (Santa Cruz, sc-8035). Unless otherwise stated, all antibodies were used at a working dilution of 1:500 for western blotting and 1:400 for IF. Secondary antibodies used are as follows: mouse Alexa Fluor 647 (Thermo Fischer Scientific, A-21235), Rabbit Alexa Fluor 647 (Thermo Fischer Scientific, A-21244), Rabbit Alexa Fluor 488 (Thermo Fischer Scientific, A-11008), Mouse Alexa Fluor 488 (Thermo Fischer Scientific, A-11029), Mouse HRP (Sigma-Aldrich, A9044) and Rabbit HRP (Sigma-Aldrich, A6154). Secondary antibodies for IF were used in dilution 1:500 and for immunoblotting in dilution 1:10000.

### Gene silencing

Commercially available SMARTpool ON-TARGET oligonucleotides targeting human eIF2AK3 (HRI) (L-005007), uL5 (RPL11) (L-013703), TP53 (L-003329) and non-targeting siRNA (D-001810) were purchased by Horizon Discovery (Dharmacon). siRNA against RPL5 (uL18) was custom-made Horizon Discovery (Dharmacon) according to [28]. Cells were transfected with 20 nM siRNA using Lipofectamine RNAiMAX reagent (Thermo Fisher Scientific, 13778150) according to the manufacturer’s instructions. After 48 h or the times indicated, cells were harvested and lysed for RNA or protein extraction.

### AgNOR staining

Cells were seeded in 8-well chambered cell culture slides (Falcon) at a density of 10^4^ cells/well. A vehicle well was included, containing cells treated with DMSO. The cells were fixed with 2% glutaraldehyde in PBS for ten min at room temperature, following two sequential washing steps with PBS and dH2O, respectively. Next, cells were incubated with a second fixing solution (methanol: acetic acid 3:1) for 5 min and washed thoroughly with dH2O. Further, 200 μL of silver nitrate colloidal solution (a 0.5 g/mL silver nitrate aqueous solution was diluted in 2% gelatin and 1% formic acid, in a proportion of 2:1) was added to each well and incubated in the dark for 20 min, at room temperature. Then, the slides were washed vigorously with dH2O, and coverslips were mounted using Prolong Gold (Invitrogen, P36930). AgNOR stained foci -which appeared as black-brown dots within the nucleus- were evaluated under a light microscope at (×20 and ×40) magnification (Olympus BX53). The photos were taken with a digital color camera (Olympus DP73).

### Cell survival assays

Cells were seeded at a density of 5000 cells/well in 96-well plates (#3904, Corning®, Sigma Aldrich) 18–24 h prior to treatment, in a total volume of 100 μL of DMEM. For the dose-response experiments, two-fold serially diluted concentrations of CuET were applied. The inhibitory effect of the CuET and its combinations was determined by measuring cell viability using the resazurin assay. After 48 h incubation with the compounds, 20 µL of resazurin working solution (0.15 mg/mL in DPBS) (#R7017, Sigma Aldrich) was added to each well, and the plates were further incubated for 2 h. The emitted fluorescence was measured with a microplate reader using the 560 nm excitation/590 nm emission filter set (Tecan Infinite M1000 Pro, Männedorf, Switzerland). The assay was performed with three technical and three biological replicates, including vehicle controls (DMSO-treated cells), negative no-cell controls, and background subtraction controls (media and compound without cells) to ensure that the tested compounds are not autofluorescent at the applied wavelength. Results were normalized as % viability compared with the vehicle control after background subtraction, using GraphPad Prism 7.0 (Graph Pad Software Inc., San Diego, CA). Single-agent dose-response curves were plotted by applying three-parametric nonlinear regression. GI_50_ values were automatically calculated by the software. Synergistic analysis and heatmap plotting were performed based on the Bliss reference model using SynergyFinder, by importing the mean %viability values to the application [31] The synergy scores calculated for each dose pair have positive and negative values denoting synergy (red) and antagonism (green), respectively.

### Preparation of cell extracts and western blotting

Following chemical treatment or transfection, subconfluent cells were lysed in RIPA buffer supplemented with a cocktail of protease and phosphatase inhibitors (Thermo Fisher Scientific, 78444) and sonicated for five cycles of 30 seconds on and 15 s off, in a Bioruptor® (Diogenode). Following lysate clearance with centrifugation for 10 min at 13000 rpm and 4 °C, protein quantitation was performed with the DC™ Protein Assay Kit II (Bio-Rad, 5000112). 20 μg of cell lysate was boiled in Laemmli sample buffer for 5 min at 95 °C, loaded onto SDS-PAGE gels, and transferred onto nitrocellulose or PVDF membranes. Chemiluminescence signal was detected using SuperSignal™ West Dura (Thermo Fisher Scientific, 34076). Images were acquired with an Amersham Imager 600 scanner. Cell fractionation was performed as previously described [2]. TCA precipitation was used for protein extraction following polysome profiling.

### Quantitative real-time PCR

Total RNA extraction was performed with PureLink™ RNA Mini Kit (Thermo Fisher Scientific (12183025), and RNA extraction from polysome profiling fractions was achieved with Trizol followed by clarification with the Norgen Biotek Corp RNA Clean Up/concentration kit (Norgen Biotek, 298-23600). Quantitative RT-PCR was conducted with TaqMan™ RNA-to-CT™ 1-Step Kit (Thermo Fisher Scientific, 4392938) in a StepOnePlus™ Real-Time PCR System (Applied Biosystems). Primers for 47S rRNA, *TP53*, *NOXA* and *BBC3* were previously described [28]. Primers: *BAX*: F, 5ʹ-GGCCCTTTTGCTTCAGGGTT-3ʹ, R 5ʹ-CTCGCTCAGCTTCTTGGTGG-3ʹ, *BAK*: F, 5ʹ ATCCCGGCAGGCTGATCC-3ʹ, R, 5ʹ-GGGCTACCTGCTCCTCAGAA-3ʹ and *BID*: F, 5ʹ- AGCTGCAGACTGATGGCAAC-3ʹ, R, 5ʹ-GGATGCTACGGTCCATGCTG-3ʹ. TaqMan probes (Thermo Fisher Scientific): beta-actin (Hs01060665_g1), *TP53* (Hs01034249_m1), *CDKN1A* (Hs00355782_m1), *MDM2* (Hs01066930_m1).

### Transcriptomics

RNA sequencing was performed by the core facility for Bioinformatics and Expression Analysis (BEA) in Karolinska, Huddinge, Sweden. Briefly, following RNA quality assessment by TapeStation electrophoresis (Agilent), libraries were prepared with Illumina RiboZero TruSeq Stranded mRNA, and samples were sequenced in a HiSeq2500 system (Illumina). Data preprocessing, performed in NGI through nf-core/RNAseq [32]. Differential expression analysis was performed using DESeq2 (v1.24.0) [33]. For downstream gene ontology (GO) analysis, DESeq2-produced data was filtered using a log2foldchange > |1| and *p* < 0.05 threshold and processed via g:Profiler [34]. Z score in GO analysis gives the likelihood that a specific GO term is increased or decreased and is described in [35]. Time-dependent analysis was performed with maSigPro [36]. Data analyses were performed in RStudio (R-4.0.3) and GraphPad Prism (v.9).

### Microscopy

Cells were seeded on 6well plates containing coverslips (Thermo Scientific™ Nunc™ Thermanox™ Coverslips, 12-565-88) or 96well plates one day prior to treatment. Following treatment, cells were washed three times with PBS, fixed in 4% formaldehyde (Sigma-Aldrich, F8775) for 10 min, and permeabilized for 10 min using cold methanol or PBS 0.5% Triton X-100 (Sigma-Aldrich, X100). They were subsequently washed three times with PBS and incubated for 1 h at room temperature in Blocking Solution (3% BSA in PBS) (Sigma-Aldrich, A7906). Fixed cells were incubated at room temperature with primary antibodies for 1 h at dilution 1:400 (unless otherwise stated), washed three times in PBS, and incubated with secondary antibodies for 1 h at room temperature (dilution 1:500). Finally, cells were stained with 10 μM Hoechst (Thermo Fisher Scientific, 62249) or DAPI (Thermo Fisher Scientific, 62249, D1306) for 30 min at room temperature and the coverslips were mounted onto slides. FBL and NPM were used as a marker for nucleolar segregation. Images were acquired using a Nikon Eclipse Ti2 inverted epifluorescent microscope. EU staining was performed with Click-iT™ RNA Alexa Fluor™ 488 Imaging Kit (Thermo Fisher Scientific, C10329) respectively, OPP staining with Click-iT™ Plus OPP Alexa Fluor™ 488 Protein Biogenesis Assay Kit (Thermo Fisher Scientific, C10456) and AHA staining with Click-iT™ AHA Alexa Fluor™ 488 Protein Synthesis Assay Kit (Thermo Fisher Scientific, C10289) according to the manufacturer’s instructions. Images were acquired using an IN Cell Analyzer 2200 (GE Healthcare) and analyzed or fluorescence microscope Zeiss LSM780 and analyzed using Cell Profiler, Fiji (Image J), and RStudio.

### Translation monitoring

For polysome profiling, cells in 70–80% confluent 15 cm dishes were washed twice in PBS containing 100 µg/ml cycloheximide (CHX) (Sigma-Aldrich, C7968) and lysed post-treatment in hypotonic buffer supplemented with CHX [37]. Lysates were cleared by centrifugation, and equal amounts (adjusted by 260 nm OD measurement) were loaded onto 5–50% sucrose gradients produced in sucrose gradient buffer with a gradient forming unit (Biocomp). Samples were centrifuged at 36000 rpm for 3 h using an SW41Ti rotor in an Optima XE-90 ultracentrifuge (Beckman-Coulter). The samples were then analyzed in a piston gradient fractionator (Biocomp) using the company’s software. Twenty-eight fractions were collected at a pace of 2.86 mm/fraction (A540 nm) and were further used for protein extraction (see relevant sections).

### Tumor tissues and immunohistochemistry

Indirect immunoperoxidase staining was carried out using formaldehyde-fixed, paraffin-embedded tissue sections from archival specimens of two well-defined cohorts of human carcinomas (see below for the patient samples). For antigen unmasking, the deparaffinized sections were boiled in a microwave oven for 15–20 min in citrate buffer (pH = 6), followed by overnight incubation with the primary antibody anti-NPLOC4 (HPA 023295, Atlas Antibodies) before initiating the staining procedure. The sensitive immunoperoxidase staining method was performed using the Vectastain Elite kit (Vector Laboratories) to detect the primary antibody and a nickel sulfate- enhancement step without nuclear counterstaining to visualize the chromogenic (diaminobenzidine) reaction [38]. The immunohistochemical staining patterns were evaluated by a senior oncopathologist and classified based on NPL4 protein localization in relation to cell nucleoli. The cohorts of human archival specimens of triple-negative breast and serous ovarian carcinomas were previously described in [39, 40]. Retrospective Triple Negative Breast Cancer (TNBC) biopsies from 63 clinically high-risk patients (high-risk definition according to the Danish Breast Cooperative Group (https://www.dbcg.dk/ accessed 22.10.2009) that underwent mastectomy between 2003 and 2015 were selected and classified as being triple negative according to the criteria set in the ASCO/CAP guidelines (ER < 1%, PR < 1%, HER2 0, 1+ or 2+ but FISH/CISH negative). The patients presented a unifocal tumor of an estimated size of more than 20 mm. None of the patients had previous surgery on the breast and did not receive preoperative treatment. This study was conducted in compliance with the Helsinki II Declaration and written informed consent was obtained from all participants and approved by the Copenhagen and Frederiksberg regional division of the Danish National Committee on Biomedical Research Ethics (KF 01-069/03). Paraffin-embedded material from the cohort of ovarian tumors was collected at the Department of Pathology, University Hospital, Las Palmas, Gran Canaria, Spain, from surgical operations performed in the period 1995–2005. For the purpose of the present study, only samples from serous ovarian carcinomas (*n* = 51) were used. The use of long-term stored tissue samples in this study was in accordance with the Spanish codes of conduct (Ley de Investigación Biomédica) and was approved by the review board of the participating institution. Patients were informed that samples may be used for research purposes under the premise of anonymity.

### Zebrafish transplantation

Animal experiments were performed in the zebrafish facility of Karolinska Institute per the national ethical guidelines and regulations (N207/14). Experiments on embryos younger than 5 days do not require an ethical permit). tdTomato/luciferase stably expressing MDA-MB-231 breast cancer cells, generated with a lentivirus-based approach (#32904, Addgene), were injected into Tg(fli:EGFP) zebrafish embryos as previously described [41]. 25 embryos were randomly allocated to each experimental condition and analyzed by a separate investigator (blinded). Embryos were imaged using ImageXpress Nano (Molecular Devices) and the tumor size was assessed with ImageJ [42]. Embryos with out-of-focus tumor images and/or different image positioning in treated-untreated conditions were considered biased and excluded from the analysis. Fish embryo acute toxicity test was performed according to the OECD guidelines Test No. 236 [43].

### Mining public databases

To study the correlation among DE genes upon CuET treatment and genes essential following disulfiram treatment, we combined our RNA-Seq analysis with RNA-Seq expression data from DepMap [21] (v.21Q3).

### Statistics and reproducibility

RNA-Sequencing experiments were performed in biological triplicates. Data normalization, modeling, and statistical testing were performed according to the corresponding differential expression analysis packages in R (DESeq, maSigPro). qRT-PCR, image, and survival analyses included a minimum of three independent biological and multiple technical replicates to reach a minimum sample size of *N* = 30 that according to the central limit theorem allows for the assumption of a normal distribution [44]. Normality was checked with the D’Agostino & Pearson test. Under these conditions, parametric unpaired t-tests or ANOVA were used for statistical significance testing. In all the other cases (*N* < 30) statistical significance analysis was performed with the Mann–Whitney (two variables) or the Kruskal–Wallis test (multiple variables). Outliers were identified with the ROUT method (Q = 1%) and excluded from statistical testing. Sample size (*N*) and *p* (or FDR) values are provided in the corresponding figures or figure legends.

## Supplementary information

Reproducibility_checklist

Supplementary_info_Revised

Supplementary_Fig_Revised

Supplementary_Uncropped_WB_Revised

Supplementary_Table_1_Revised

## Data Availability

All data generated or analyzed during this study are included in this published article and its supplementary information files. The RNA-Seq FASTQ files generated specifically for this study can be accessed from the NCBI’s BioProject repository using the IDs: PRJNA777552.
