# Supplementary material for: Actionable cancer vulnerability due to translational arrest, p53 aggregation and ribosome biogenesis stress evoked by the disulfiram metabolite CuET
Source: Cell Death Differ. 2023 May 4;30(7):1666–78. doi: 10.1038/s41418-023-01167-4 (PMC10307793; doi:10.1038/s41418-023-01167-4)
Supplement: Supplementary file 2 — Supplementary_info_Revised [file 41418_2023_1167_MOESM2_ESM.docx]

**Supplementary material**

**i) Uncropped Western Blot images**

**ii) Table S1**

Table S1a: DE genes (2hrs CuET Vs DMSO), produced in R with DESeq2

Table S1b: DE genes (6hrs CuET Vs DMSO), produced in R with DESeq2

Table S1c: Pearson correlation coefficients between gene expression and Disulfiram efficacy (DepMap, 21Q2, PRISM 19Q4)

Table S1d: Gene assignment in 6 clusters (following DE analysis in R with MaSigPro)

Table S1e: Reactome pathway enrichment analysis of upregulated genes (g:Profiler)

Table S1f: Reactome pathway enrichment analysis of downregulated genes (g:Profiler)

Table S1g: DE genes (16hrs thapsigargin Vs DMSO), produced in R with DESeq2

Table S1h: Reactome pathway enrichment analysis of DE genes from S1g (g:Profiler)

Table S1i: Reactome pathway enrichment analysis of DE genes from S1b (g:Profiler)

Table S1j: Toxicity assay in zebrafish for either CuET or AQ alone or their combination. All tested concentrations were well tolerated with no observed death.

**ii) Figures S1-S5**

**Figure legends**

**Supplementary Fig. 1.** **CuET inhibits translation**. **A** Representative IF images of TIAR in U2OS cells following treatment with CuET or thapsigargin (1.5hr). Scale bar = 10 μm. **B** Click-IT O-propargyl-puromycin (OPP) incorporation in A549 cells treated with CuET or ActD^L^. Scale bar: 50 µm **C** Click-IT L-Azidohomoalanine (AHA) incorporation in U2OS cells treated with CuET, CHX, or the p97 chemical inhibitor NMS-873. Scale bar = 20 μm. ΝPL4

**Supplementary Fig. 2. Nucleolar reformation following CuET treatment**. **A** Quantitation of the nucleolin and 5.8S rRNA levels (IF) in A549 cells treated with CuET or ActD^L^ for the indicated time points. 750-1500 cells were analyzed per experiment (data shown as mean ± SD, n = 3 biological replicates, **p < 0.01). Scale bar: 50µm. **B** Expression levels of 47S rRNA in A549 treated with CuET or DMSO vehicle (non-significant values are not shown). **C** Representative IF images of MDA-MB-231 cells depicting NPL4 and FBL (as a nucleolar marker) protein localization following CuET, ActD^L^ or BMH-21 treatment. Scale bar: 2 μM. **D** Co-immunoprecipitation of RPL5 (uL18) and MDM2 in cells treated with CuET or ActD^L^ for the indicated time points. **E** p53 and p21 protein levels following CuET or ActD^L^ treatment +/- knock-down of RPL5 (uL18) or RPL11 (uL5) in A549 cells.

**Supplementary Fig. 3. NPL4 is essential for p53 entrapment in aggregates and its neutralization. A** Immunoblotting of p53 levels in cells treated with CuET for various time points. **B** Immunoblotting of NPL4, p53, MDM2 and CHK2 protein levels in U2OS cells treated with CuET and fractionated in the supernatant (S/N) or insoluble (pellets) parts. Lamin B and α-tubulin were used as markers for the soluble and insoluble fractions, respectively. **C** Representative IF images of GFP-tagged NPL4 and p53 protein levels following ectopic expression of NPL4^mut^  in U2OS cells stably expressing a DOX-inducible NPL4-GFP cassette (16hrs DOX). Scale bar: 10 μM. **D** Immunoblotting of p53 after treatment of A549 cells with CuET +/- chemical inhibitors against ATM, ATR or DNA-PK. **E** Phosphorylated p53 (ser15) protein levels following treatment of A549 cells with Neocarzinostatin or chemical inhibitors against ATM and DNA-PK. Numbers below the blot indicate the signal ratio p-p53/β-actin. **F** Protein levels of phosphorylated CHK1 (ser317) in A549 cells treated with hydroxyurea (HU) or a chemical inhibitor against ATR. Numbers below the blot indicate the signal ratio p-CHK1/β-actin. **G** Immunoblotting of various p53 post-translational modifications (PTM) in A549 cells treated with CuET +/- Etoposide (3 hours). Etoposide was used as a positive control known to induce activating p53 PTMs. **H** *MDM2* mRNA levels following treatment of A549 cells with CuET (6 hours) +/- Etoposide (last 2 hours) (data shown as mean ± SD, n = 3 biological replicates, **** p < 0.001, *** p = 0.01, ** p < 0.01, non-significant values are not shown).

**Supplementary Fig. 4. CuET alters the transcriptomic landscape of A549 cells.** **A** Average IC50 values in WT and mutant p53 cancer cell lines (data retrieved from ^2^). **B** Scatterplot depicting the association among Achilles R coefficients (DepMap) and log2(fold expression) of DE genes following CuET treatment. The purple oval highlights the most important associations. **C** Graphic illustration of the RNA-Seq experimental design. **D** Scatterplot showing the accumulated expression kinetics of DE genes following treatment of A549 cells with CuET for 2 or 6 hours. Six clusters were observed based on the expression patterns. **E**, **F** Cell cycle staging in A549 (E) or RPE1 (F) following treatment with either 0.1μM CUET or 10μM Bleomycin for 24 hours. The analysis was based on EdU incorporation and Hoehst immunostaining. 1000-2000 cells were analysed per experiment (data shown as mean ± SD, n = 3 biological replicates, **** p < 0.001, ** p < 0.01, * p < 0.05, non-significant values are not shown).

**Supplementary Fig. 5.** **Inhibiting RiBi or autophagy enhances the anti-survival activity of CuET.**

**A** Dose-response viability assay and GI_50_ shift calculation of U2OS cells treated with either CuET, BMH21, or the combinations of CuET with BMH-21 (upper) (data shown as mean ± SD, n = 3 biological replicates). GI_50_ values are shown in the table (lower) with 95% Confidence intervals along with R^2^ values quantifying the goodness of fit). **B** Dose-response viability assay and GI_50_ shift calculation of U2OS cells treated with CuET +/- CX-5461. **C-D** Synergy plots of CuET and BMH-21 (C) or CuET and CX-5461 (D) upon 48-hour treatment with a 3x3 concentration matrix, measuring cell viability. Synergy plots (left) show the Bliss synergy score obtained for each combination across the matrix, indicating the synergistic areas in red and the antagonistic in green. **E** IC50 Dose-response viability assay and GI_50_ shift calculation of U2OS cells treated with either CuET, CQ or their combination. **F** Synergy plot of CuET and CQ **G** Dose-response viability assay and GI_50_ shift calculation of U2OS cells treated with either CuET, AQ, or their combination. **H** Synergy plot of CuET and AQ. **I** Quantitation of survival rates following a 48-hour treatment of RPE1 cells with the synergistic pairs of either CuET and CQ. **J** Quantitation of the click-OPP signal in RPE1 and A549 cells treated with 1μM CuET for 1 hour. **K** NPL4 protein accumulation shown by western blotting in RPE1 or U2OS cells treated with the indicated concentrations of CuET (3 hours).
