# Supplementary figures and images for: Actionable cancer vulnerability due to translational arrest, p53 aggregation and ribosome biogenesis stress evoked by the disulfiram metabolite CuET
Source: Cell Death Differ. 2023 May 4;30(7):1666–78. doi: 10.1038/s41418-023-01167-4 (PMC10307793; doi:10.1038/s41418-023-01167-4)

Figure S1.

A.

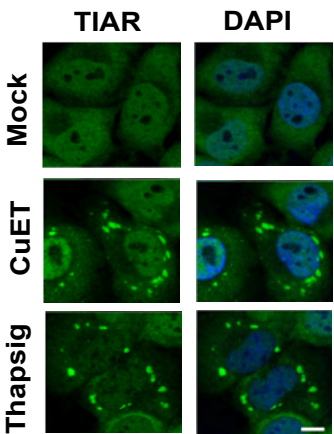

B.

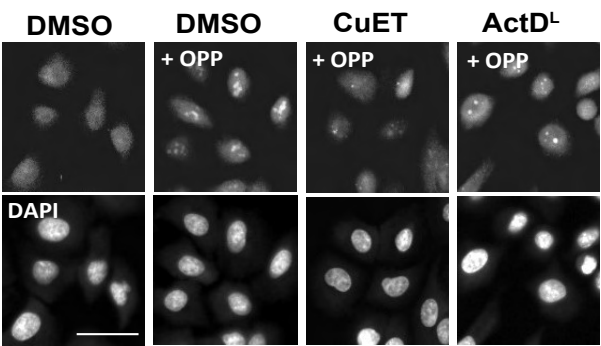

C.

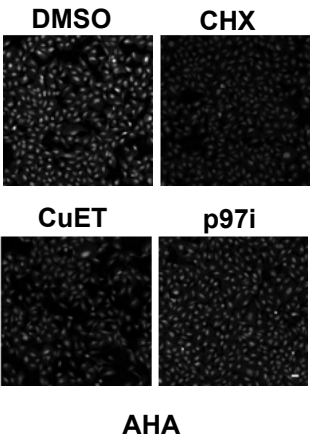

Figure S2

A.

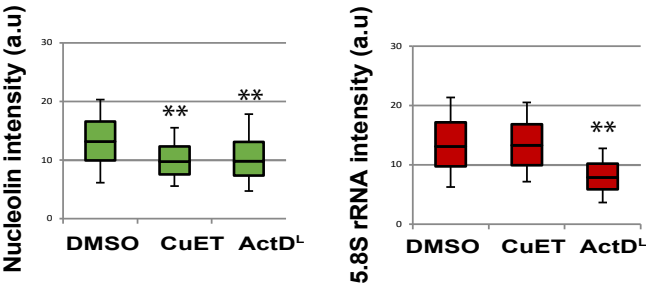

B.

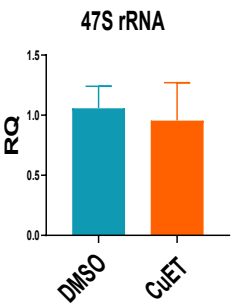

C.

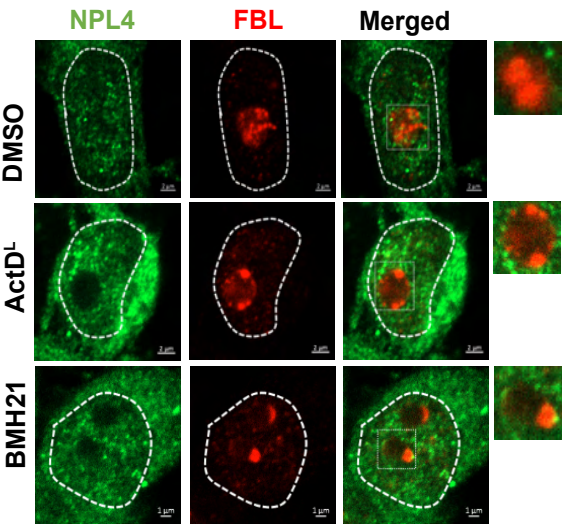

D.

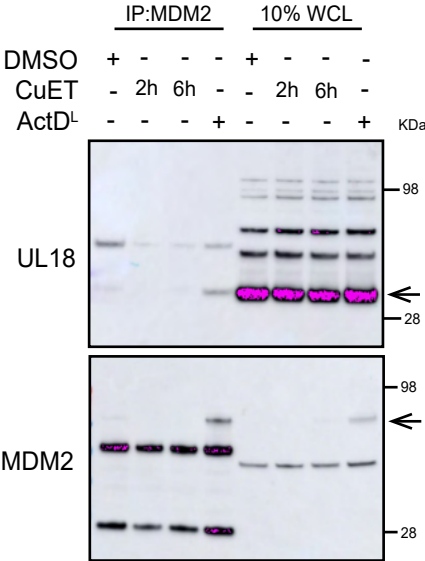

E.

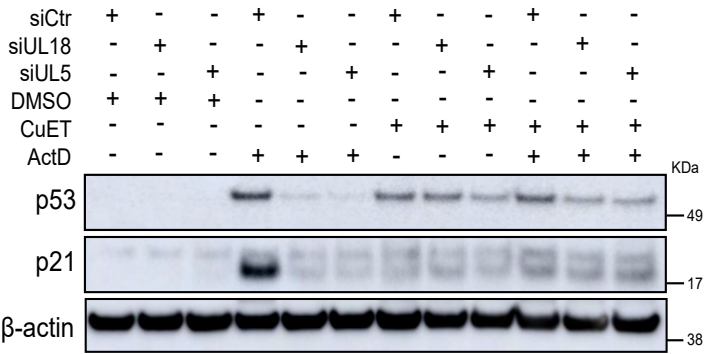

Figure S3

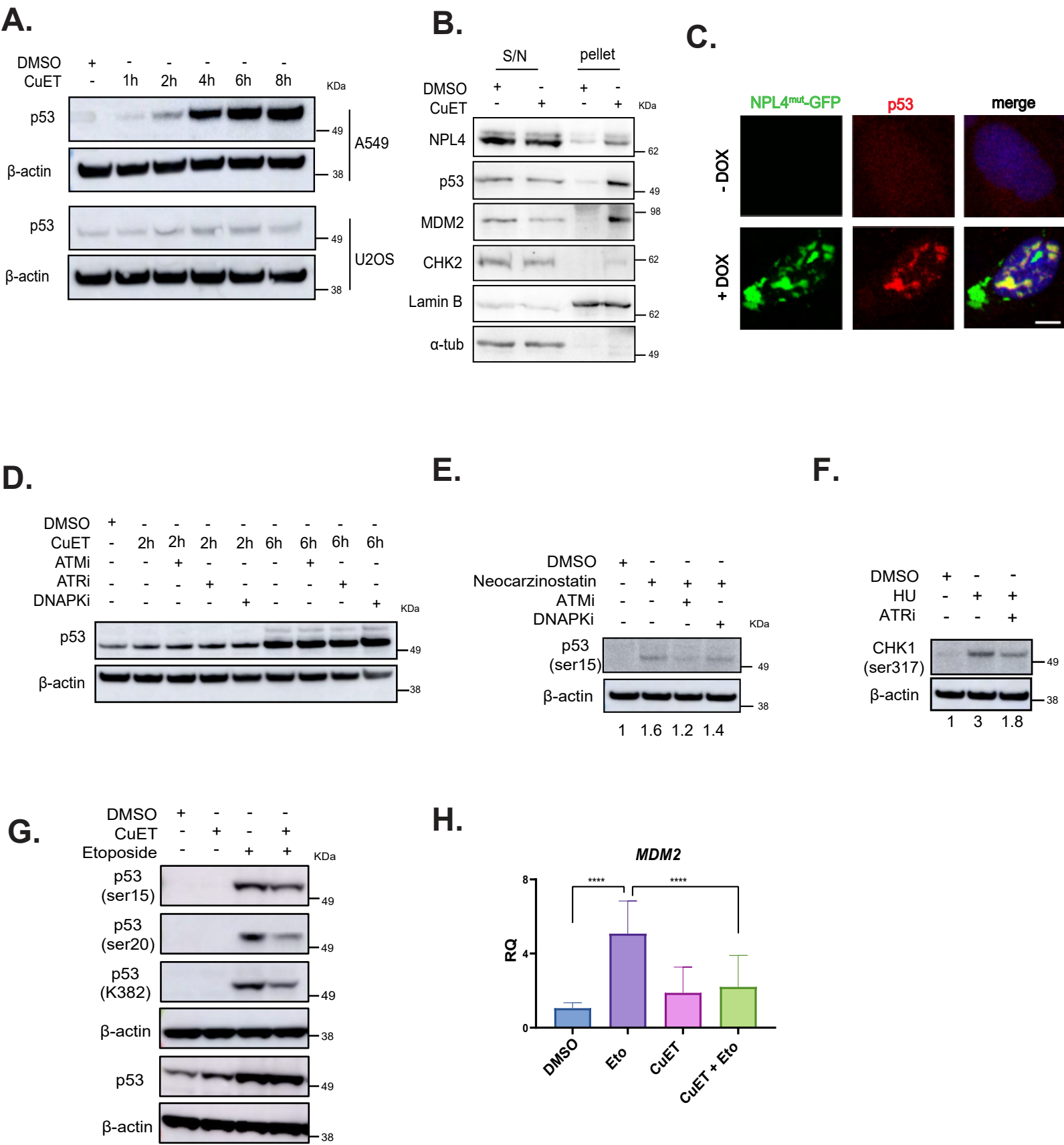

Figure S4.

A.

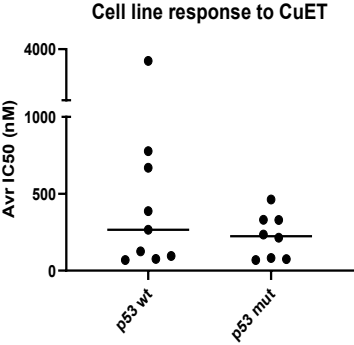

B.

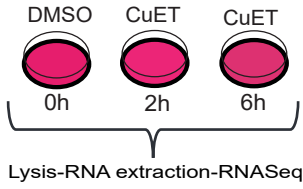

C.

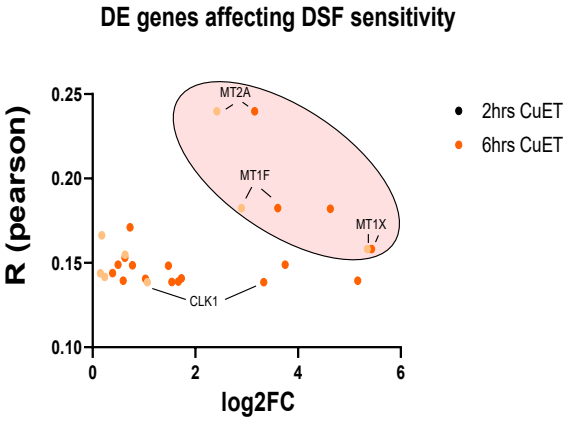

D.

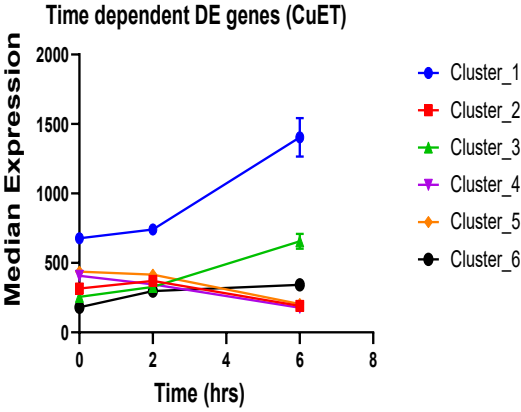

E.

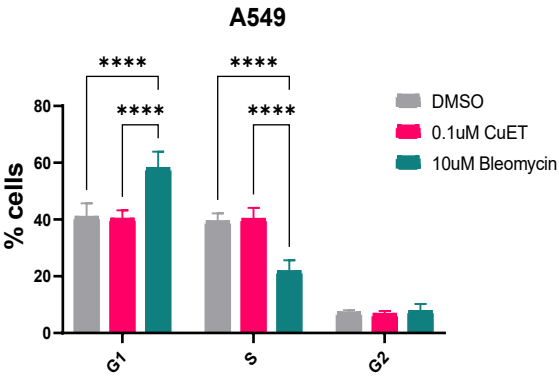

F.

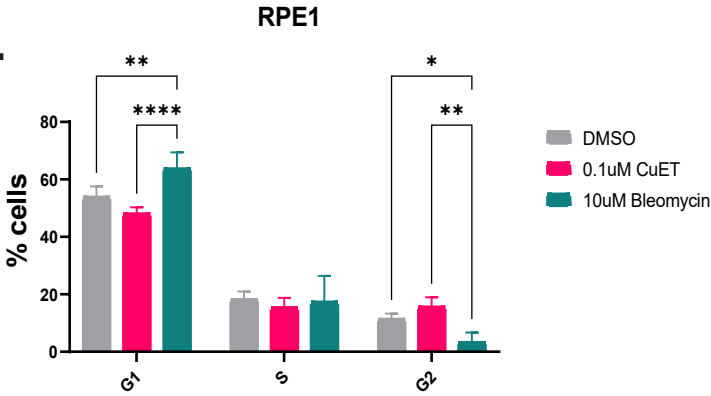

Figure S5

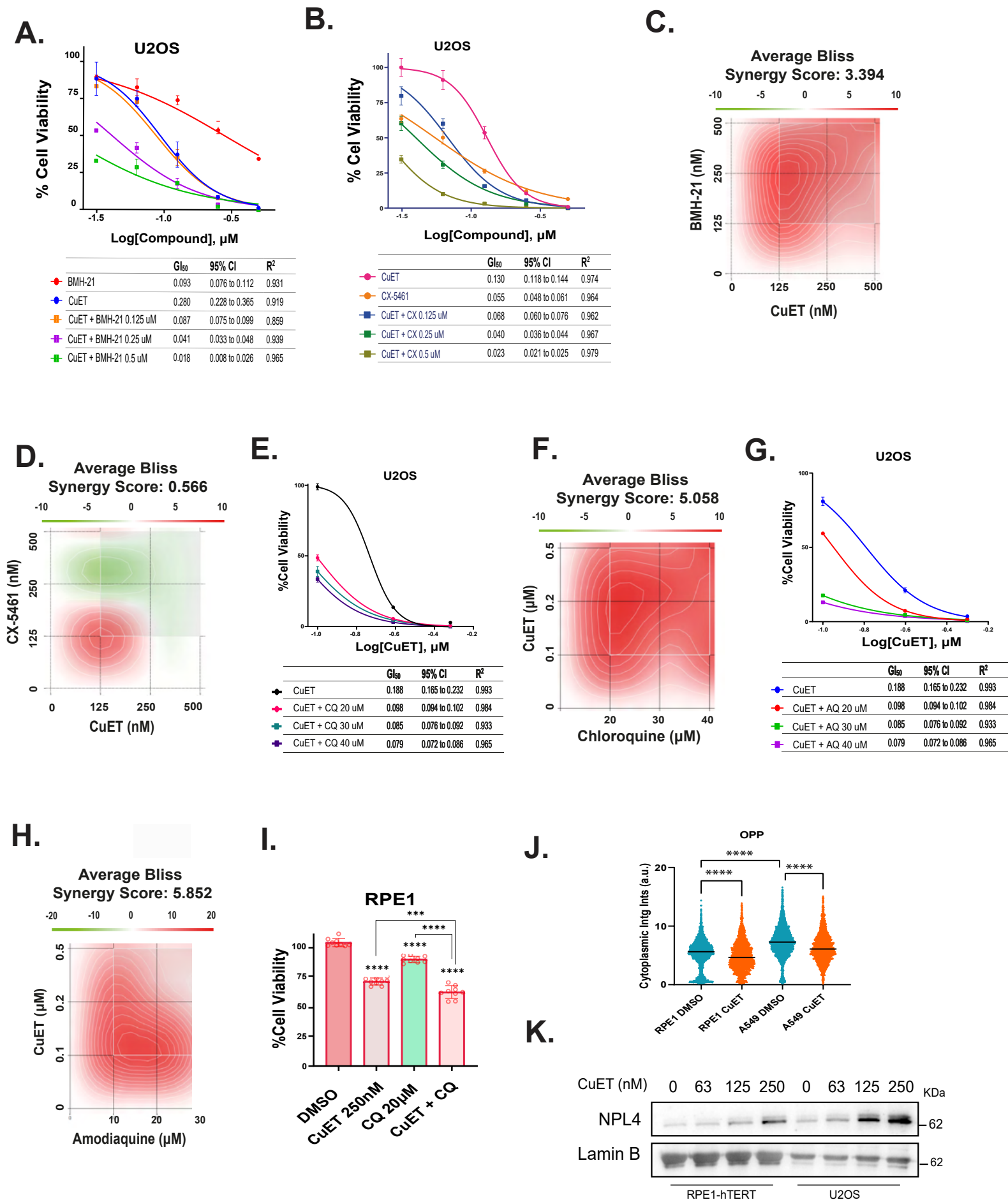

Supplement: Supplementary file 3 — Supplementary_Fig_Revised [file 41418_2023_1167_MOESM3_ESM.pdf]
