# Supplementary material for: Actionable cancer vulnerability due to translational arrest, p53 aggregation and ribosome biogenesis stress evoked by the disulfiram metabolite CuET
Source: Cell Death Differ. 2023 May 4;30(7):1666–78. doi: 10.1038/s41418-023-01167-4 (PMC10307793; doi:10.1038/s41418-023-01167-4)

Uncropped WB images.1

Figure 1A.

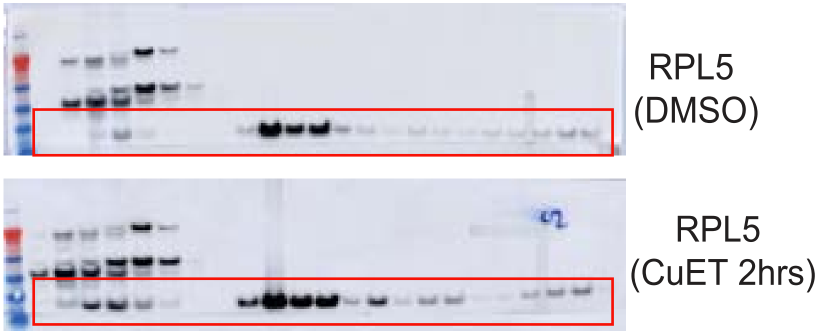

Figure 1D.

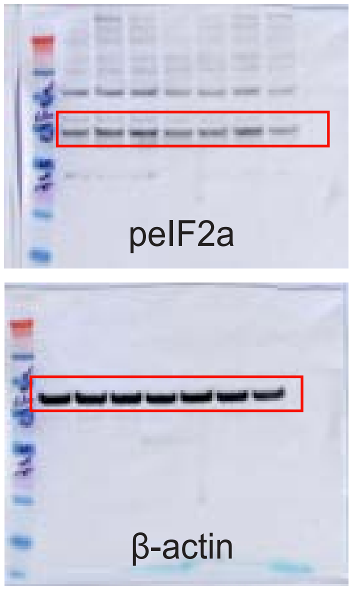

Figure S2D.

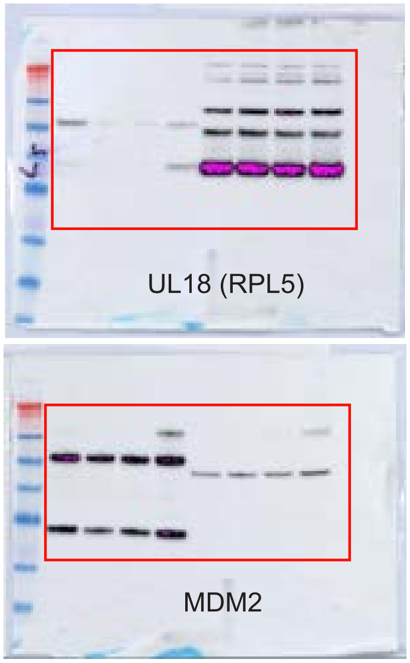

Figure S2E.

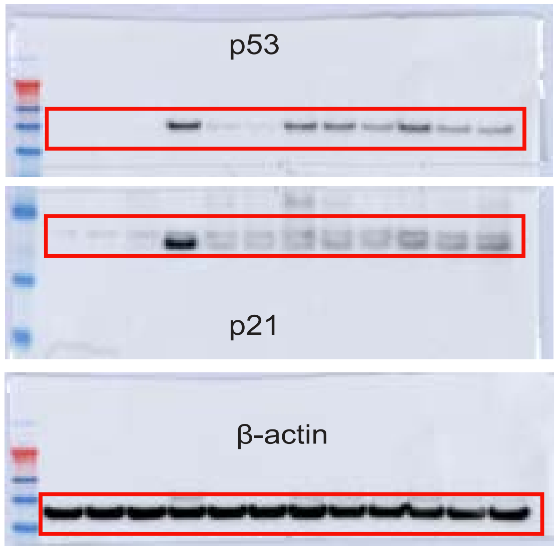

Figure 3A.

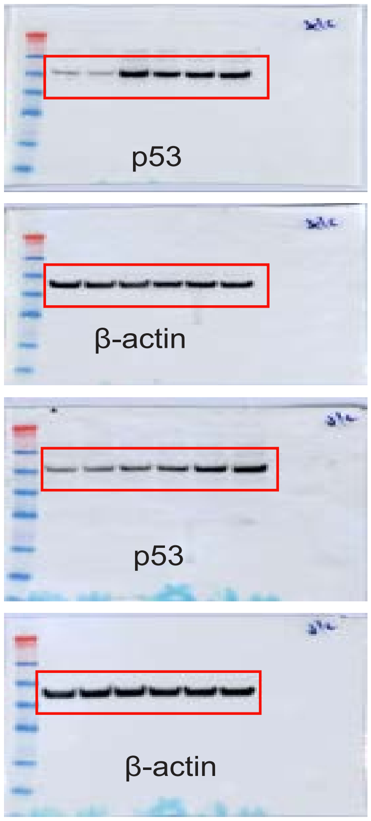

Uncropped WB images.2

Figure 3C.

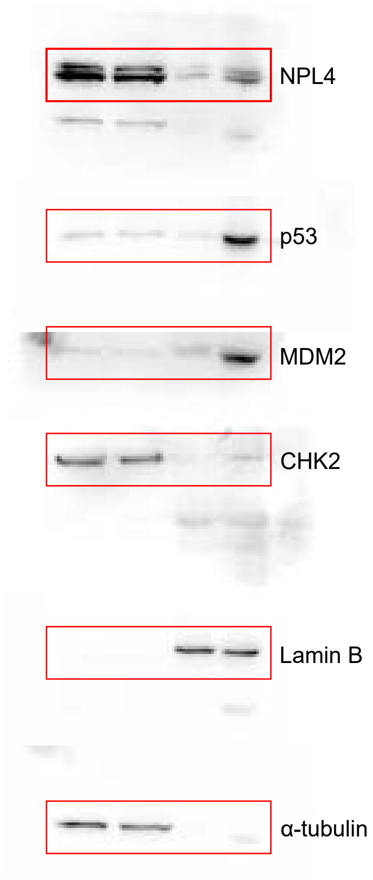

Figure 3E.

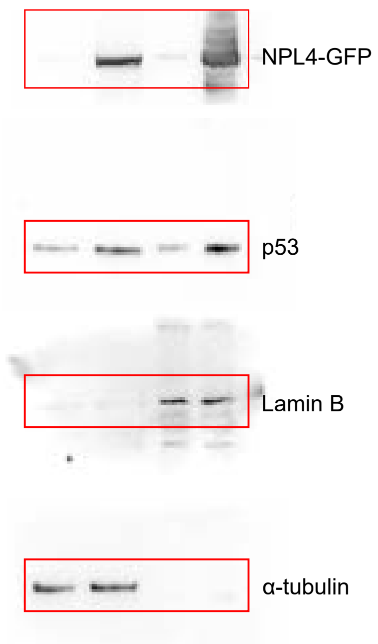

Figure 3F.

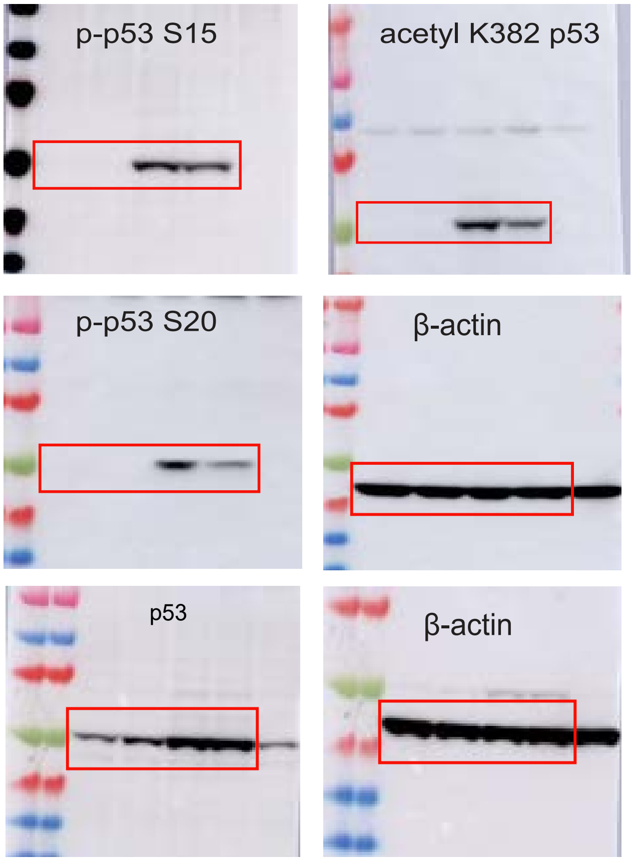

Figure S3A.

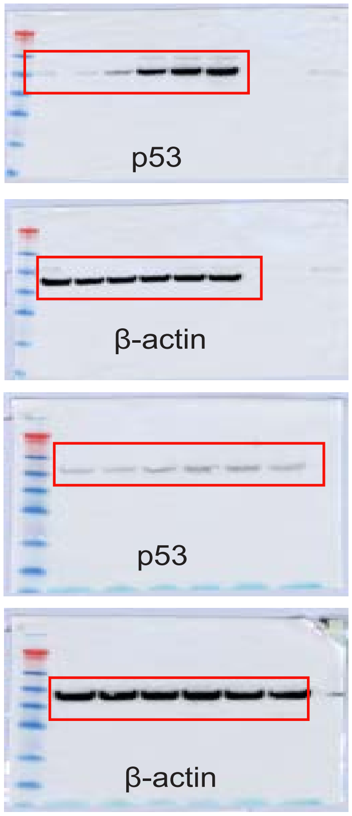

Figure S3B.

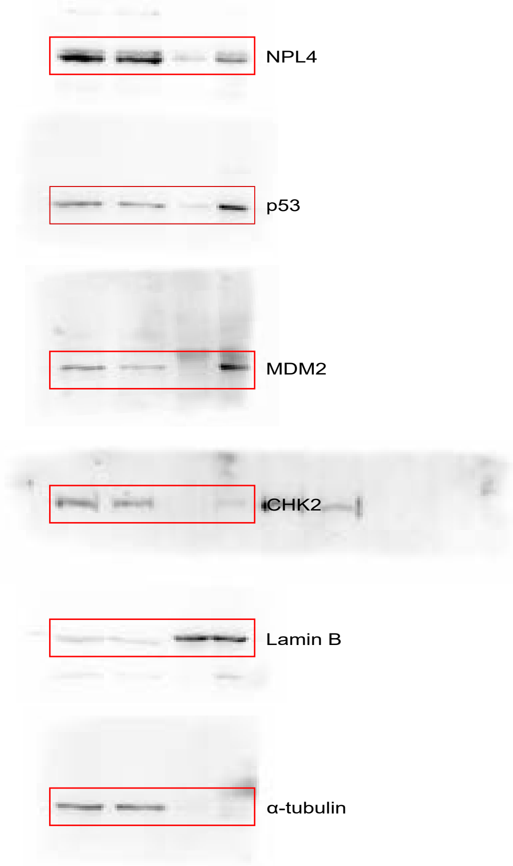

Figure S3D.

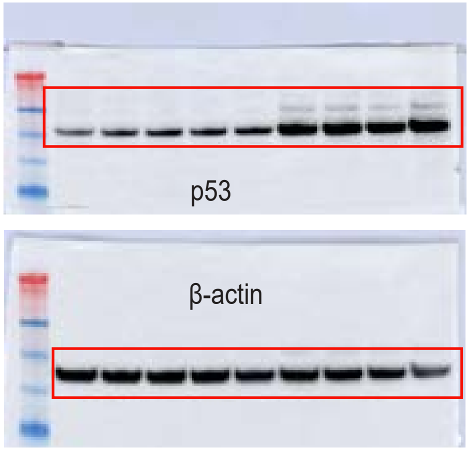

Uncropped WB images.3

Figure S3E,F.

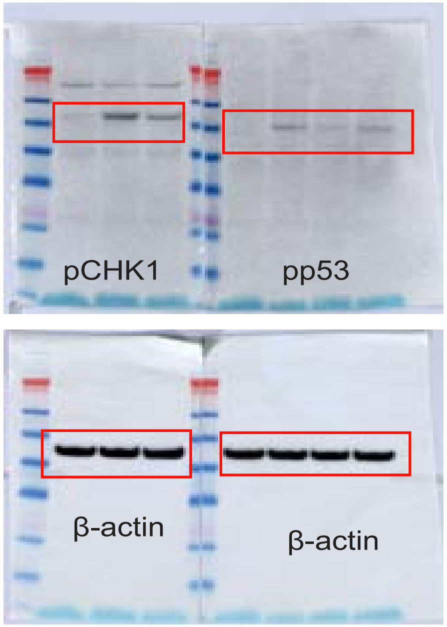

Figure S3G.

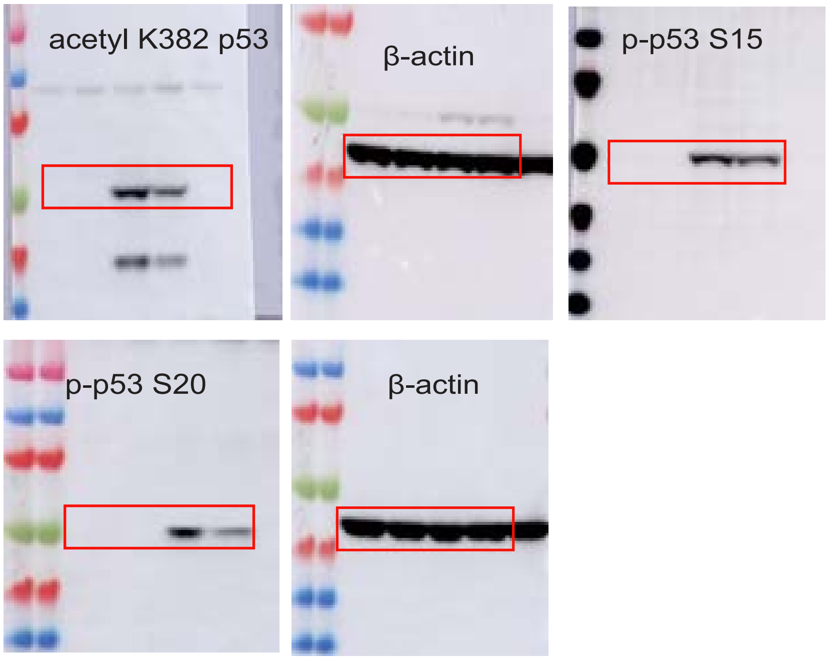

Figure S5K.

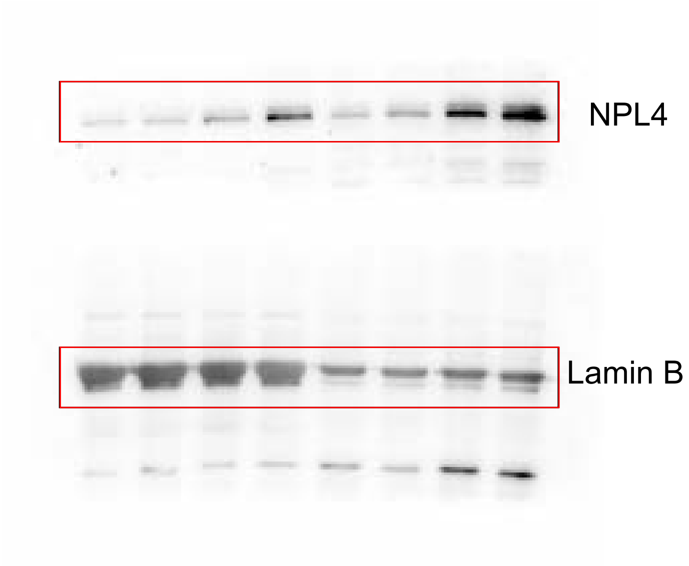

Supplement: Supplementary file 4 — Supplementary_Uncropped_WB_Revised [file 41418_2023_1167_MOESM4_ESM.pdf]
